# Radiofrequency Ablation versus Cryoablation in the Treatment of Paroxysmal Atrial Fibrillation: A Meta-Analysis

**DOI:** 10.1155/2018/6276241

**Published:** 2018-04-01

**Authors:** Ali H. Hachem, Joseph E. Marine, Housam A. Tahboub, Sana Kamdar, Shaffi Kanjwal, Ronak Soni, Khalil Kanjwal

**Affiliations:** ^1^Central Michigan University, College of Medicine, Mt. Pleasant, MI, USA; ^2^John's Hopkins Institute, Baltimore, MD, USA; ^3^Mclaren Cardiovascular Group, Michigan State University, East Lansing, MI, USA

## Abstract

**Background:**

Pulmonary vein isolation is commonly performed using radiofrequency energy with cryoablation gaining acceptance. We performed a meta-analysis of randomized controlled trials which compared radiofrequency versus cryoablation for patients with atrial fibrillation.

**Methods:**

A systematic search strategy identified both published and unpublished articles from inception to November 10, 2016, in multiple databases. The primary outcomes for this meta-analysis were long-term freedom from atrial fibrillation at 12-month follow-up and overall postoperative complication rates. For all included studies, the methodological quality was assessed through the Cochrane Collaboration's tool for risk of bias.

**Results:**

A total of 247 articles were identified with eight being included in this review as they satisfied the prespecified inclusion criteria. Overall, there was no significant difference in freedom from atrial fibrillation at ≥12-month follow-up between those receiving cryoballoon and radiofrequency ablation, respectively (OR = 0.98, CI = 0.67–1.43, *I*^2^ = 56%, *p*=0.90). Additionally, the secondary outcomes of duration of ablation, fluoroscopy time, and ablation time failed to reach significance. Cryoballoon ablation had significantly greater odds of postoperative phrenic nerve injury at 12-month follow-up.

**Conclusions:**

Our meta-analysis suggests that cryoballoon ablation provides comparable benefits with regard to freedom from atrial fibrillation at medium-term follow-up, fluoroscopy time, ablation time, operative duration, and overall complication rate in comparison to radiofrequency ablation.

## 1. Background

Atrial fibrillation (AF) is a common arrhythmia affecting up to 2% of the general population [1, 2]. AF commonly affects the elderly and is associated with a 26% lifetime risk in males and 23% lifetime risk in females by age 80 [3]. Patients with AF have increased mortality and increased risk of stroke. In addition to that, there is an increased risk of various cardiac complications, including myocardial ischemia/infarction and congestive heart failure exacerbation and dementia [4].

The first line therapy for AF includes the use of pharmacological agents, aiming at rate control, rhythm control, and anticoagulation; however, the recurrence rate with antiarrhythmic drugs is still high and side effects of these medications require close monitoring [4, 5]. The standard treatment for patients with paroxysmal drug refractory AF is catheter ablation via pulmonary vein isolation. Catheter ablation has emerged as the most effective rhythm control strategy. Catheter ablation is differentiated by the energy source used. The two current modes of energy being used are radiofrequency and cryothermal energy [4].

Radiofrequency ablation (RFA) causes tissue damage using heat while cryoablation causes tissue damage by freezing the target region. RFA has been considered to be the standard technique and is utilized more frequently than cryoablation. However, it is time consuming, requires extensive training, and is associated with an increased risk of cardiac perforation, thromboembolism, and pulmonary vein stenosis [5–7]. Cryoablation, using a cryoballoon catheter, is gaining acceptance as being equally as effective as RFA with potentially decreased rates of complications, although there has been a higher reported rate of phrenic nerve palsy [5, 8, 9].

While catheter ablation is the mainstay for treatment of AF, the optimal method of ablation is currently under debate. Prior meta-analyses on the topic have been conducted, but they were either inconclusive or included a wide array of study designs, thereby limiting their overall quality and external validity. The overall goal of this meta-analysis is to compare the long-term effectiveness, as measured by freedom from atrial fibrillation at 12-month follow-up, and the complications in treating adult patients (≥18 years old) with atrial fibrillation, using these two methods.

## 2. Methods

### 2.1. Criteria of Study Inclusion

All studies that randomly allocated adult patients (≥18 years old) to receive either cryoballoon or radiofrequency ablation for atrial fibrillation were considered for eligibility. Authors of studies for which the randomization sequence or generation tool was unknown were contracted for clarification. Finally, quasi-randomized studies were also considered for eligibility if there was mention of a quasi-random method of allocation such as by date, case number, or date alteration. Nonrandomized studies were excluded to limit overall heterogeneity and improve internal validity of the results.

### 2.2. Search Methods for Identification Studies

A search strategy was created to identify both published and unpublished articles from inception to May 10, 2016, in the following databases: MEDLINE, Embase, Cochrane Library, and the Database of Abstracts of Review of Effects (DARE). The full search strategy can be viewed in Table 1; however, briefly, the terms of the search related to cryoballoon, catheter ablation, radiofrequency, and cryoablation. The results generated by the search strategy were reviewed and screened. The initial agreement between the reviewers based on full-text eligibility was assessed through the calculation of an unweighted kappa (*κ*). As per the Cochrane guidelines, a *κ* value can quantitatively assess the agreement between two reviewers [10]. As such, values between 0.40 and 0.59 were considered to represent fair agreement, those between 0.60 and 0.74 represented good agreement, and those that were 0.75 or higher represented excellent agreement [8].

### 2.3. Primary and Secondary Outcomes

The primary outcomes for this meta-analysis were long-term freedom from atrial fibrillation at 12-month follow-up and overall postoperative complication rates. The secondary outcomes for this review were procedure time, fluoroscopy time, and ablation time.

### 2.4. Data Management and Extraction

For studies that satisfied the inclusion criteria for this meta-analysis, the data was extracted as per a standardized data extraction form. The data extraction form was first created and piloted by an independent reviewer. The form was used to collect information related to the location of where the study was performed, the various follow-up times, the data reported for the prespecified outcomes, and any miscellaneous outcome information that was not intended to be analyzed by our review. Any data that was reported exclusively in graphical form was extracted using a graph-digitizing software (GraphClick, Arizona Software).

### 2.5. Assessment of Methodological Quality and Risk of Bias

The Cochrane Collaboration's tool for assessing risk of bias [10] was used by two independent reviewers to evaluate the methodological quality of each included trial. This tool included questions related to randomization, blinding, and outcome data reporting. For each question, the risk of bias was reported as low risk, unclear risk, or high risk of bias. The initial agreement between the reviewers was assessed through the calculation of an unweighted *κ*. Again, *κ* values between 0.40 and 0.59 were considered to represent fair agreement, those between 0.60 and 0.74 represented good agreement, and those that were 0.75 or higher represented excellent agreement [10]. In certain situations, the authors of included articles were contacted to elaborate on the methodology used during their research. This was done in order to obtain additional information and to ensure an accurate quality assessment.

### 2.6. Statistical Analyses and Measurement of Treatment Effect

The primary outcomes of freedom from atrial fibrillation at 12-month follow-up and overall complications are dichotomous variables. As such, an odds ratio (OR) with a 95% confidence interval (CI) was calculated for these outcomes.

On the other hand, all secondary outcomes were measured in units of time. All time measures were first standardized to a total in minutes. In situations where a median and interquartile range (IQR) were reported, statistical conversions were made to a mean and standard deviation (SD) using the methods described by Wan et al. [11]. In situations where a mean and confidence interval (CI) were reported, statistical conversions were made to a mean and SD using the methods described by the Cochrane Collaboration [12]. Overall, a mean difference (MD) in the unit of minutes with a 95% CI was calculated for all secondary outcomes.

In situations where data could be pooled, a meta-analysis was performed using the Mantel–Haenszel random-effects model since there was expected heterogeneity between the included studies. *p* values less than 0.05 were considered to be significant. For continuous outcomes, an MD value less than 0 represented a decrease in value in the specific outcome (e.g., time of procedure, ablation time, or fluoroscopy time) when cryoballoon was used. On the other hand, an MD value greater than 0 represented a gain in value of the specific variable with cryoballoon use.

### 2.7. Assessment of Heterogeneity

To calculate heterogeneity, an *I*^2^ statistic test was used. The threshold for conducting subgroup analyses was an *I*^2^ greater than 40%. As suggested by the Cochrane Handbook for Systematic Reviews, an *I*^2^ greater than 40% suggests that heterogeneity may be present [10]. If heterogeneity was present, a priori subgroup analysis was performed on the basis of overall study quality and type of radiofrequency ablation used (Duty Cycle Radiofrequency Ablation versus Irrigated Radiofrequency Energy). Subgroups were only created if more than two studies fell into a specific subgroup. Sensitivity analysis was performed by sequentially removing studies in which a different type of radiofrequency ablation was used.

### 2.8. Assessment of Publication Bias

A funnel plot was created for the primary outcomes and was visually inspected to assess for publication bias. As per the Cochrane guidelines, in the absence of bias, the plot should generally take the shape of a symmetrical, inverted funnel.

### 2.9. Data Management

All forest and funnel plots were generated using Review Manager software (RevMan version 5.2; Nordic Cochrane Centre, Cochrane Collaboration). Agreement between the reviewers, as assessed through the unweighted *κ*, was calculated using SPSS software (version 21.0; SPSS Inc.). Finally, all tests of significance were two-tailed and *p* values less than 0.05 were considered significant.

## 3. Results

### 3.1. Study Inclusion

**Figure 1:**
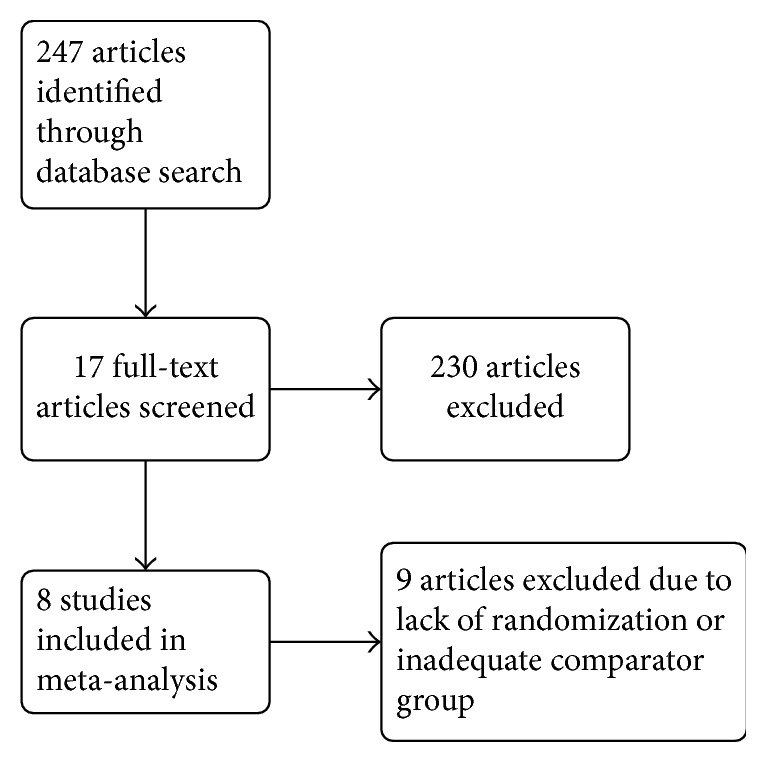
Flow diagram of study inclusion.

A total of 247 articles were identified by the systematic literature search. After an initial title and abstract screening, a total of 17 were considered and retrieved to assess their eligibility by review of the full text. Of these articles, a total of eight satisfied the prespecified inclusion criteria and were included in this review [5, 6, 13–18]. A flow diagram of study inclusion can be viewed in Figure 1. Articles were excluded if they were nonrandomized or lacked an adequate comparison group. The raw agreement between the independent reviewers for full eligibility was 88.2% and the unweighted *κ* was calculated to be 0.75, which represents good agreement between the two reviewers.

### 3.2. Study Characteristics

A detailed description of all the included studies can be viewed in Table 2. A total of eight studies encompassing 1548 patients undergoing either cryoballoon or radiofrequency ablation were included. All of the included studies were published between the years 2012 and 2015. Studies were conducted at centers across continental Europe, the United Kingdom, and Russia. Seven of the included studies had published study protocols available for review [5, 6, 13–17]. Seven of the studies compared cryoballoon with irrigated radiofrequency energy ablation [5, 6, 13–17], whereas one made the comparison with phased duty-cycled radiofrequency energy ablation [18].

### 3.3. Risk of Bias Assessment

**Figure 2:**
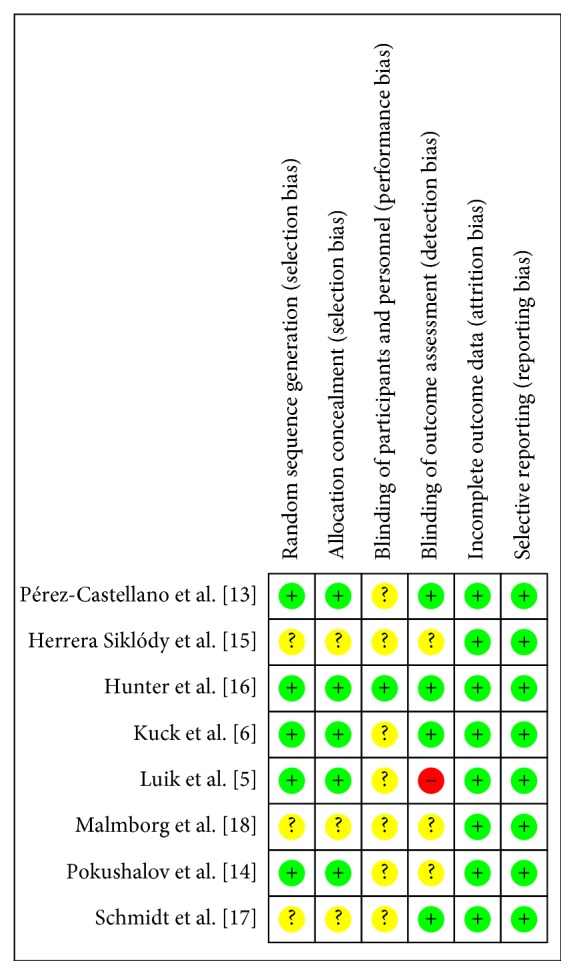
Risk of bias of included studies in the meta-analysis.

**Figure 3:**
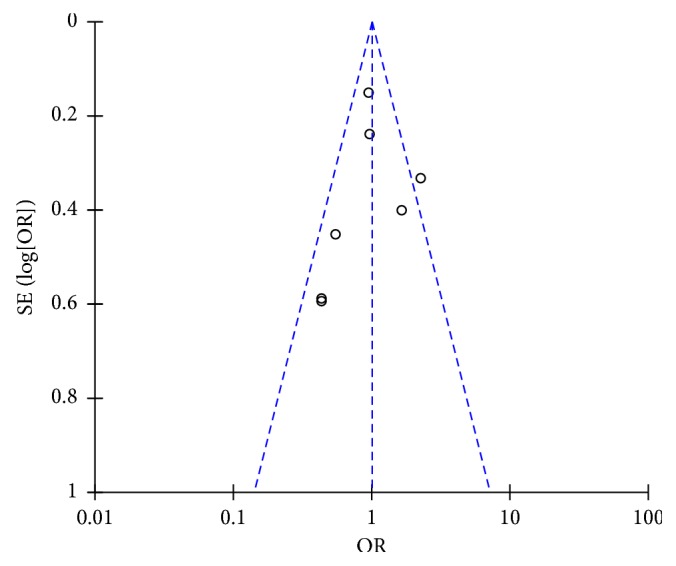
Funnel plot for assessment of publication bias for freedom from atrial fibrillation at 12-month follow-up.

**Figure 4:**
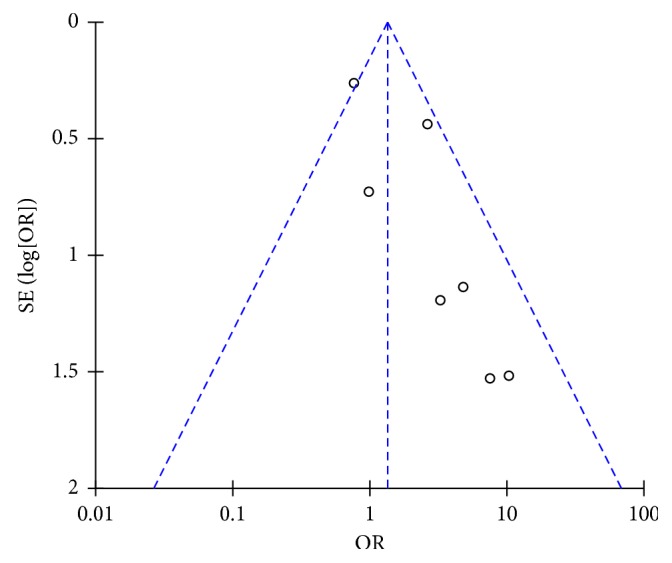
Funnel plot for overall postoperative complication rate.

The majority of studies had a low risk or unclear risk of bias for several methodological parameters as assessed through the Cochrane tool for risk of bias assessment (Figure 2). Only one study sufficiently reported information on the blinding of study participants and personnel [16]. Visual inspection of the funnel plot for all primary outcomes (freedom from atrial fibrillation and overall complication rate) did not suggest publication bias; however, there were few studies with large effect sizes (Figures 3 and 4). Thus, publication bias cannot be entirely ruled out.

### 3.4. Freedom from Atrial Fibrillation at Long-Term Follow-Up

**Figure 5:**
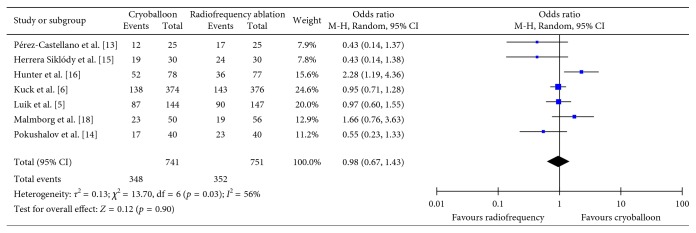
Mean odds ratio with 95% CI of patients free from atrial fibrillation at ≥12-month follow-up.

A total of eight studies assessed freedom from atrial fibrillation at ≥12-month follow-up (*n*=1542) [5, 6, 13–18]. Across these studies, 53% (393/741) of patients receiving cryoballoon ablation and 53% (399/751) of patients receiving radiofrequency ablation developed atrial fibrillation at ≥12-month follow-up. Overall, it was found that there was no significant difference in freedom from atrial fibrillation at ≥12-month follow-up between those who received cryoballoon ablation and those who received radiofrequency ablation (OR = 0.98, CI = 0.67–1.43, *I*^2^ = 56%, *p*=0.90) (Figure 5). These results were robust to sensitivity analysis as the removal of one study which utilized phase duty-cycled radiofrequency ablation did not alter the results (OR = 0.90, CI = 0.60–1.36, *I*^2^ = 58%, *p*=0.62).

### 3.5. Procedure Duration

**Figure 6:**
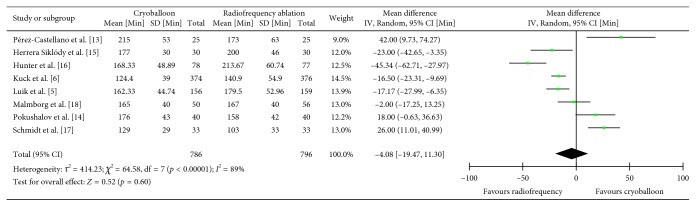
Mean procedure time in minutes with 95% CI of patients receiving cryoballoon versus radiofrequency ablation.

Eight studies assessed the procedure time (*n*=1582) [5, 6, 13–18]. The weighted mean procedure duration using cryoballoon ablation and radiofrequency ablation was found to be 161.79 minutes in comparison to 165.87 for radiofrequency ablation. Overall, cryoballoon ablation was found to be 4.08 minutes shorter; however, this difference failed to reach significance (MD = −4.08, CI = −19.47 –11.30, *I*^2^ = 89%, *p*=0.60) (Figure 6). These findings were robust to sensitivity analysis as nonsignificance remained (MD: −4.20, CI = −21.75–13.36, *I*^2^ = 91%, *p*=0.64) even after removal of the data from the one study that utilized phase duty-cycled radiofrequency ablation.

### 3.6. Fluoroscopy Time

**Figure 7:**
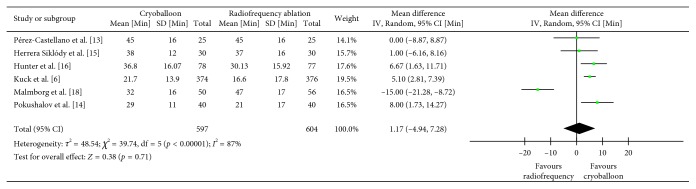
Mean fluoroscopy time in minutes with 95% CI in patients receiving cryoballoon versus radiofrequency ablation.

A total of six studies assessed fluoroscopy time (*n*=1204) [6, 13–16, 18]. The weighted mean fluoroscopy time was found to be 33.11 minutes with cryoballoon ablation and 31.94 minutes with radiofrequency ablation. Although using cryoballoon ablation prolonged fluoroscopy time by 1.17 minutes, this result failed to reach significance when compared to radiofrequency ablation (MD = 1.17, CI = −4.94–7.28, *I*^2^ = 87%, *p*=0.71) (Figure 7). Sensitivity analysis revealed a significant prolongation of fluoroscopy time by 5.07 minutes when the data from the phased duty-cycled ablation was removed and cryoballoon was compared with irrigated radiofrequency energy ablation in isolation (MD = 5.07, CI = 3.21–6.93, *I*^2^ = 0%, *p* < 0.00001).

### 3.7. Ablation Time

**Figure 8:**
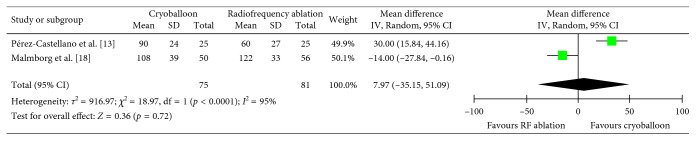
Mean ablation time in minutes with 95% CI in patients receiving cryoballoon versus radiofrequency ablation.

Two studies assessed ablation time (*n*=155) [13, 18]. The weighted mean ablation time was found to be 99.02 minutes with cryoballoon ablation and 91.06 minutes with radiofrequency ablation. Overall, although ablation time was 7.97 minutes longer with cryoballoon ablation, no significant difference was observed when compared with radiofrequency ablation (MD = 7.97, CI = −35.15–51.09, *I*^2^ = 95%, *p*=0.72) (Figure 8). Sensitivity and subgroup analysis was not performed due to the limited number of studies in this outcome.

### 3.8. Postoperative Complications

**Figure 9:**
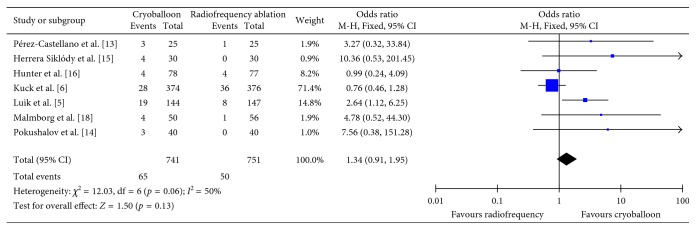
Mean odds ratio of overall postoperative complications with 95% CI in patients receiving cryoballoon versus radiofrequency ablation.

Seven studies reported complications associated with both radiofrequency and cryoballoon ablation (*n*=1492) [5, 6, 13–16, 18]. A list of all complications reported by the included studies in this meta-analysis can be viewed in Table 3. Irrespective of the type of complication and the transient nature of the complication, cryoballoon ablation was found to have a 1.90 times greater odds of having a complication in comparison to radiofrequency ablation; however, this difference failed to reach significance (OR = 1.90, CI = 0.88–4.11, *I*^2^ = 50%, *p*=0.10) (Figure 9). These findings were unaltered by sensitivity analysis (OR = 1.73, CI = 0.77–3.89, *I*^2^ = 53%, *p*=0.18).

We further stratified the overall complication rate based on the type of complication reported. Overall, it was found that cryoballoon ablation had a 10.3 times greater odds of postoperative phrenic nerve injury at 12-month follow-up in comparison to radiofrequency ablation alone (OR = 10.3, CI = 3.09 to 34.6, *I*^2^ = 0%, *p*=0.0001) [5, 6, 14–16, 18]. Five of these studies reported that phrenic injury was transient and resolved at 12-month follow-up [5, 14–16, 18]; one study reported permanent phrenic nerve injury in one patient [6]. In regards to cardiac tamponade, the odds of this complication did not significantly differ between cryoballoon and radiofrequency ablation [5, 6, 16] (OR = 0.39, CI = 0.11–1.40, *I*^2^ = 0%, *p*=0.15). A nonsignificant difference in risk favoring cryoballoon was also seen with the outcome of postoperative atrial flutter [6, 13, 15] (OR = 0.63, CI = 0.10 to 4.15, *I*^2^ = 57%, *p*=0.63).

## 4. Discussion

In the present meta-analysis, we found no difference in the freedom from atrial fibrillation at 12 months between patients randomized to cryoballoon and those randomized to radiofrequency catheter ablation of atrial fibrillation. These results are consistent with recently published data from the Fire and ICE study [6]. Although patients undergoing cryoballoon ablation had shorter total procedure duration when compared to RF ablation by a mean of 4 mins, this difference did not reach statistical difference in the hands of experienced operators. RF ablation requires point-by-point circumferential ablation around pulmonary veins and may be more time consuming and technically challenging for new operators.

The fluoroscopy time was prolonged in cryoballoon ablation group by a mean of 1.17 minutes, but this small difference was not statistically significant. We found that there was a higher risk of postprocedure atrial flutter in the RF group, which was not significant.

There was a significantly higher incidence of phrenic nerve injury with cryoablation; however, most of these injuries resolved at 12 months. Thus, there was no difference in the risk of persistent phrenic nerve injury at 12 months. Phrenic nerve injury is a common concern during cryoballoon ablation of AF. Although the risk of transient phrenic nerve injury was almost ten times higher in cryoballoon group, only one patient in one study had persistent injury at 12 months of follow-up. Thus, the risk of permanent phrenic injury was low in the cryoballoon group as well.

Pulmonary vein isolation using RF energy utilizes point-by-point wide circumferential lesion. This wide area ablation can sometimes result in creating a substrate for postablation atrial flutter. Potentially, RF ablation should be associated with higher risk of postablation atrial flutter; however, in our analysis, the risk of atrial flutter was not significantly different in the two groups.

In this analysis, we found no significant differences in the overall success, fluoroscopy times, total ablation times, procedure times, and any complications seen in the two groups. Cryoballoon has become increasingly used over last few years due to several potential advantages. The need of a single transseptal puncture for cryoballoon is a definite advantage, although some centers perform RF ablation using a single transseptal catheterization as well. The learning curve for cryoablation may also be shorter.

Our analysis provides some important results, but several important questions remain unanswered. In recent years, force-sensing catheters have been used increasingly for RF ablation. These catheters provide real-time information about the catheter contact with the tissue and thus may improve the effectiveness of delivered RF lesions. We have no data systematically comparing force-sensing RF catheters verses cryoballoon ablation. A recent study showed no differences in the long-term outcomes when contact force information was available to the operators [19]. Our analysis included studies including both first- and second-generation cryoballoon. There was only one multicenter randomized study comparing radiofrequency ablation with contact force sense catheters with second-generation cryoballoon and found no difference in the outcomes following ablation using either strategy [20]. We also do not have information about patients in whom cryoballoon ablation was attempted but PVI was either not achieved or not attempted because of unsuitable anatomy.

The fluoroscopy times were found to be higher in the cryoballoon group. Although not statistically significant, this difference may become more relevant in the future as more centers are utilizing advanced mapping technology coupled with intracardiac echocardiography to minimize or eliminate the use of fluoroscopy during RF ablation. There is a need for devising strategies to minimize the use of fluoroscopy in patients undergoing cryoballoon ablation in the future. Monitoring for recurrence of atrial fibrillation after ablation was performed either using an implantable loop recorder or a Holter monitor for two to seven days at 3, 6, and 12 months of the follow-up.

## 5. Conclusions

In conclusion, the results of this meta-analysis reveal similar overall success rates at 12 months and comparable fluoroscopy and procedural times and long-term complications between patients undergoing cryoballoon and RF ablation for AF ablation. Given similar outcomes, operators should choose AF ablation technology based on patient-specific features and preferences as well as operator experience and preference.

## Figures and Tables

**Table 1:** Search strategies.

	*Embase*
1	cryoablation/(4285)
2	cryoballoon^∗^.mp. (1009)
3	cryoballoon^∗^.mp. (99)
4	Cryosurgery/(10,075)
5	cryosurg^∗^.mp. (11,090)
6	cryoablat^∗^.mp. (5709)
7	or/1-6 (16,355)
8	radiofrequency ablation/(21,784)
9	catheter ablation/(25,682)
10	(radiofrequency^∗^ adj2 ablat^∗^).mp. (31,386)
11	(catheter adj2 ablat^∗^).mp. (29,066)
12	(electric adj2 ablat∗).mp. (55)
13	or/8–12 (49,731)
14	atrial fibrillation/(12,529)
15	afib.mp. (668)
16	auricular fib^∗^.mp. (1107)
17	atrial fib^∗^.mp. (85,496)
18	or/14-17 (86,631)
19	7 and 13 and 18 (966)
20	remove duplicates from 19 (945)
21	random^∗^.mp. (1250,429)
22	randomized controlled trial/(403,270)
23	randomization/(70,306)
24	exp “clinical trial (topic)”/(192,249)
25	clinical trial^∗^.mp. (1213,928)
26	single blind procedure/(22,062)
27	double blind procedure/(130,675)
28	((singl^∗^ or doubl^∗^ or tripl^∗^ or trebl^∗^) adj3 (blind^∗^ or mask^∗^)).mp. (239,618)
29	experimental trial^∗^.mp. (2390)
30	or/21–29 (2054,944)
31	20 and 30 (174)

	*Medline*
1	Cryoballoon^∗^.mp. (375)
2	Cryosurgery/(11,490)
3	cryosurg^∗^.mp. (12,315)
4	cryoballoon^∗^.mp. (10)
5	Cryoablation^∗^.mp. (2508)
6	or/1–5 (13,174)
7	Catheter Ablation/(24,682)
8	(radiofrequency^∗^ adj2 ablat^∗^).mp. (13,686)
9	(electric^∗^ adj2 ablat^∗^).mp. (244)
10	(catheter adj2 ablat^∗^).mp. (27,099)
11	or/7–10 (30,238)
12	Atrial Fibrillation/(40,331)
13	AFIB.mp. (181)
14	atrial fib^∗^.mp. (57,253)
15	auricular fib^∗^.mp. (1555)
16	or/12-15 (57,440)
17	6 and 11 and 16 (446)
18	remove duplicates from 17 (441)
19	randomized controlled trial/(416,221)
20	Randomized Controlled Trials as Topic/(103,492)
21	Random Allocation/(86,790)
22	controlled clinical trial/(90,701)
23	experimental trial^∗^.mp. (2040)
24	Single-Blind Method/(21,827)
25	Double-Blind Method/(135,430)
26	((singl^∗^ or doubl^∗^ or tripl^∗^ or trebl^∗^) adj3 (blind^∗^ or mask^∗^)).mp. (196,284)
27	randomized controlled trial.pt. (416,221)
28	clinical trial.pt. (500,017)
29	rct^∗^.mp. (26,020)
30	random^∗^.mp. (1028,478)
31	exp clinical trial/(737,112)
32	clinical trial^∗^.mp. (852,720)
33	exp Clinical Trials as Topic/(292,379)
34	or/19–33 (1567,877)
35	18 and 34 (73)

**Table 2:** Study characteristics.

Study	Country	Sample size	Mean age (SD)	Intervention (sample size)	Comparison	Mean follow-up time	Outcomes assessed
Pérez-Castellano et al. [13]	Spain	50	CB = 58 (45, 62) IRF = 56 (40, 61)	Irrigated radiofrequency energy (*n*=25)	Cryoballoon (*n*=25)	12 months	(i) Freedom from atrial fibrillation, ablation time, procedure time, fluoroscopy time, complications
Herrera Siklódy et al. [15]	France	60	CB = 57 (8) IRF = 56 (10)	Irrigated radiofrequency energy (*n*=30)	Cryoballoon (*n*=30)	12 months	(i) Freedom from atrial fibrillation, procedure time, fluoroscopy time, complications, biochemical response
Pokushalov et al. [14]	Russia	80	CB = 56 (9) IRF = 56 (11)	Irrigated radiofrequency energy (*n*=40)	Cryoballoon (*n*=40)	12 months	(i) Freedom from atrial fibrillation, procedure time, fluoroscopy time, complications
Hunter et al. [16]	United Kingdom	234	CB = 56 (11) IRF = 61 (12) Combined = 58 (12)	Irrigated radiofrequency energy (*n*=77) Combined (*n*=79)	Cryoballoon (*n*=78)	12 months	(i) Freedom from atrial fibrillation, procedure time, fluoroscopy time, complications
Schmidt et al. [17]	Germany	99	CB = 66 (10) IRF = 63 (10) LB = 65 (8)	Irrigated radiofrequency energy (*n*=33) Laser balloon (*n*=33)	Cryoballoon (*n*=33)	Postoperative	(i) Asymptomatic cerebral lesions procedure time
Kuck et al. [6]	Germany	750	CB = 59.9 (9.8) IRF = 60.1 (9.2)	Irrigated radiofrequency energy (*n*=376)	Cryoballoon (*n*=374)	1.5 years	(i) Recurrence of atrial fibrillation, procedure time, fluoroscopy time, complications
Luik et al. [5]	Germany	215	CB = 61 (54, 66) IRF = 60 (54, 67)	Irrigated radiofrequency energy (*n*=159)	Cryoballoon (*n*=141)	12 months	(i) Freedom from atrial fibrillation, complications
Malmborg et al. [18]	Sweden	110	CB = 59 (9) PDRF = 62 (7)	Phased duty-cycled radiofrequency energy (*n*=56)	Cryoballoon (*n*=54)	12 months	(i) Freedom from atrial fibrillation, ablation time, procedure time, fluoroscopy time, complications, quality of life

**Table 3:** Reported postoperative complications after cryoballoon ablation versus radiofrequency ablation.

Study	Cryoballoon ablation		Radiofrequency ablation	
	Complication	Number of patients (*n*=741)	Complication	Number of patients (*n*=751)
Pérez-Castellano et al. [13]	Hemoptysis	1	Right femoral arteriovenous fistula	1
Herrera Siklódy et al. [15]	Phrenic nerve injury	2		
	Femoral pseudoaneurysm	1		
	Groin hematoma	1		
Hunter et al. [16]	Phrenic nerve injury	4	Tamponade	1
			Hematoma	1
			Asymptomatic pulmonary vein stenosis	1
			Dressler's syndrome	1
Kuck et al. [6]	Phrenic nerve injury	10	Groin site complication^∗^	16
	Groin site complication^∗^	7	Cardiac tamponade/pericardial effusion	5
	Other, nonarrhythmia complications^∗∗^	3	Pulmonary complication	4
	Pulmonary complication	2	Transient neurological complication	3
	Cardiac tamponade/pericardial effusion	1	Dyspnea	2
	Transient neurological complication	1	Gastrointestinal complication	2
	Dyspnea	1	Contrast media reaction	1
	Anxiety	1	Contusion	1
	Esophageal ulcer	1	Hematuria	1
	Gastrointestinal complication	1	Local edema	1
Luik et al. [5]	Phrenic nerve injury	9	Vascular	5
	Vascular	8	Pericardial effusion	3
	Pericardial effusion	2		
Malmborg et al. [18]	Groin hematoma	2	Groin hematoma	1
	Phrenic nerve injury	2		
Pokushalov et al. [14]	Phrenic nerve injury	3		
Total patients		65		50

^∗^As per the study, groin site complications included vascular pseudoaneurysm, arteriovenous fistula, device-related infection, hematoma, puncture site hemorrhage, and groin pain. ^∗∗^As per the study, other cardiac complications included atrial septal defects, coronary artery disease, and pericarditis.
